# Increased proportions of outdoor feeding among residual malaria vector populations following increased use of insecticide-treated nets in rural Tanzania

**DOI:** 10.1186/1475-2875-10-80

**Published:** 2011-04-09

**Authors:** Tanya L Russell, Nicodem J Govella, Salum Azizi, Christopher J Drakeley, S Patrick Kachur, Gerry F Killeen

**Affiliations:** 1 Ifakara Health Institute, Biomedical and Environmental Thematic Group, P.O. Box 53, Ifakara, Tanzania; 2 Liverpool School of Tropical Medicine, Vector Group, Pembroke Place, Liverpool, L3 5QA, UK; 3 The University of Queensland, School of Population Health, Australian Centre for Tropical and International Health, Brisbane, 4006, Australia; 4 London School of Hygiene and Tropical Medicine, Department of Infectious and Tropical Diseases, Keppel Street, London, WC1E 7HT, UK; 5 Centers for Disease Control and Prevention, Division of Parasitic Diseases and Malaria, Atlanta, Georgia, USA

## Abstract

**Background:**

Insecticide-treated nets (ITNs) and indoor residual spraying (IRS) represent the front-line tools for malaria vector control globally, but are optimally effective where the majority of baseline transmission occurs indoors. In the surveyed area of rural southern Tanzania, bed net use steadily increased over the last decade, reducing malaria transmission intensity by 94%.

**Methods:**

Starting before bed nets were introduced (1997), and then after two milestones of net use had been reached-75% community-wide use of untreated nets (2004) and then 47% use of ITNs (2009)-hourly biting rates of malaria vectors from the *Anopheles gambiae *complex and *Anopheles funestus *group were surveyed.

**Results:**

In 1997, *An. gambiae *s.l. and *An. funestus *mosquitoes exhibited a tendency to bite humans inside houses late at night. For *An. gambiae *s.l., by 2009, nocturnal activity was less (*p *= 0.0018). At this time, the sibling species composition of the complex had shifted from predominantly *An. gambiae s.s*. to predominantly *An. arabiensis*. For *An. funestus*, by 2009, nocturnal activity was less (*p *= 0.0054) as well as the proportion biting indoors (*p *< 0.0001). At this time, *An. funestus s.s*. remained the predominant species within this group. As a consequence of these altered feeding patterns, the proportion (mean ± standard error) of human contact with mosquitoes (bites per person per night) occurring indoors dropped from 0.99 ± 0.002 in 1997 to 0.82 ± 0.008 in 2009 for the *An. gambiae *complex (*p *= 0.0143) and from 1.00 ± <0.001 to only 0.50 ± 0.048 for the *An. funestus *complex (*p *= 0.0004) over the same time period.

**Conclusions:**

High usage of ITNs can dramatically alter African vector populations so that intense, predominantly indoor transmission is replaced by greatly lowered residual transmission, a greater proportion of which occurs outdoors. Regardless of the underlying mechanism, the residual, self-sustaining transmission will respond poorly to further insecticidal measures within houses. Additional vector control tools which target outdoor biting mosquitoes at the adult or immature stages are required to complement ITNs and IRS.

## Background

Millennia of co-evolution between humans, mosquitoes and malaria parasites have resulted in a highly specialized and efficient system for malaria transmission in Africa [1]. The principal African malaria vectors from the *Anopheles gambiae *complex and the *Anopheles funestus *group feed almost exclusively indoors at night, on sleeping humans [2-4]. This highly specialized feeding behaviour led to the development of effective front-line vector control tools-insecticide-treated nets (ITNs) and indoor residual spraying (IRS)-that target insecticide to human habitations [2,4,5]. Wide-spread use of ITNs, which reduces the density, feeding frequency and survival of mosquitoes at the population level by killing mosquitoes with insecticide or blocking their contact with humans [6-9], can protect all community members, even those not using a net [10-13]. In recent years, the number of success stories associated with wide-scale ITN use has increased and the incidence of malaria has begun to decline in many parts of Africa [14-16].

As the international community has now prioritized national and regional elimination with a long-term ultimate goal of malaria eradication [17], the need to understand the biological implications of wide-spread and long-term ITN use is paramount. Understanding the ecological and epidemiological characteristics of residual malaria transmission will be essential to adjust intervention strategies, as frontline tactics shift importance from primary to presently-secondary sources of transmission as the programme approaches elimination. The current study is a retrospective analysis, examining the impact of prolonged and wide-spread ITN pressure on the feeding patterns of anopheline mosquito populations in East Africa. Shifts in vector feeding patterns to avoid intra-domicilary vector control tools would be accompanied by shifts in the importance of various sources of transmission; for example, mosquito populations feeding more outside at dusk or dawn would be responsible for proportionally more transmission events. It was hypothesized that wide-scale ITN use would precipitate a change in the vector population, investigating possible shifts in human-biting time or location over a 12-year period.

## Methods

### Study area

The study was conducted in the Kilombero Valley (8.1°S and 36.6°E) in south-eastern Tanzania. The communities experience hyper-endemic malaria transmission [18], with a peak during the main rainy season (March - May), when larval habitat area expands. The primary vectors are from the *Anopheles gambiae sensu lato *(s.l.) complex, which is represented by two behaviourally distinctive species: *An. gambiae sensu stricto *(s.s.) and *Anopheles arabiensis *[19]. A third, locally important vector species is *An. funestus *s.s., which belongs to the *An. funestus *group.

**Figure 1:**
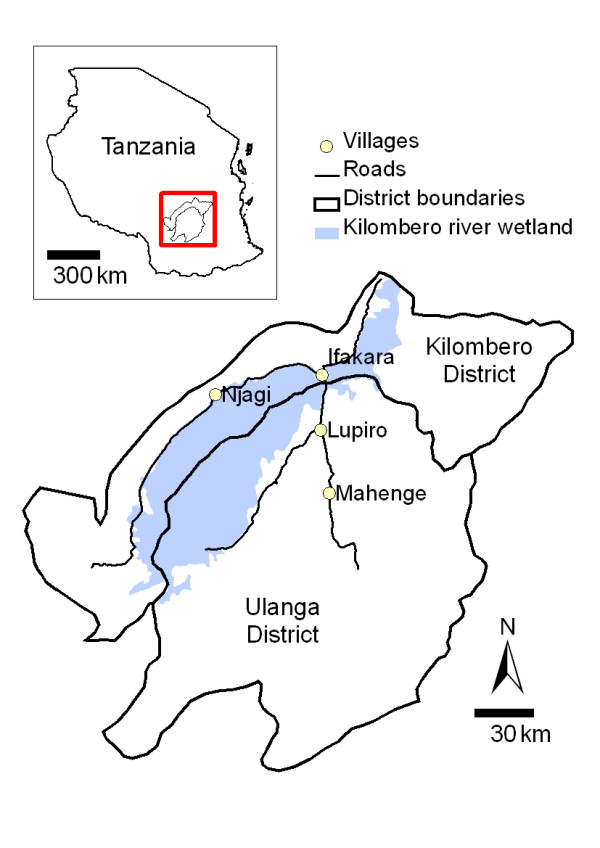
**Kilombero and Ulanga districts (8.1°S and 36.6°E) in Tanzania showing Njagi and Lupiro villages**.

The ecosystem of the Kilombero Valley is dominated by a low lying river valley, 150 km long and up to 40 km wide, which is inter-dispersed with villages and rice farms. During the current study, mosquitoes were collected from two villages (Njagi and Lupiro), both situated on the valley floor and with similar ecosystems (Figure 1). Recent genetic research indicates mosquito population structuring in the Kilombero Valley is associated with ecological complexity, opposed to geographical distance. Thus, mosquito samples from Njagi and Lupiro villages would represent one interbreeding, genetically homogenous population of *An. gambiae *s.l., even though the villages are situated on opposing sides of the Kilombero Valley [20].

A successful cost-sharing scheme for subsidising and promoting bed nets and home insecticide treatment kits was initiated in 1999 in an effort to alleviate the malaria burden [18] and this scheme was eventually adopted at national level in 2000 [21], resulting in steadily increasing coverage of nets and then insecticide treatments over the last decade [22]. While no ITN surveys were conducted in 1997 (when the first entomological survey was conducted), by the start of the first valley-wide ITN promotion scheme in 1999, demographic surveillance system (DSS) surveys of >60,000 people revealed that <10% of people owned a bed net [18]. Subsequent surveys through the same DSS platform were conducted annually to estimate usage of both untreated and treated nets [22].

### Study design

The indoor and outdoor biting profiles of *An. gambiae *s.l. and the *An. funestus *group were estimated using human landing catches (HLC) in Nov 1997, Jul/Aug 2004 and May/Jun 2009. The estimates for 1997 represent mosquito behaviour before ITN use became wide-spread, 2004 was after 75% community-wide use of untreated bed nets had been achieved and 2009 was after 47% use of ITNs and 91% use of any net had been reached a year previously [22]. The 1997 vector behaviour survey was conducted in Njagi village [23,24] whereas the 2004 and 2009 surveys were conducted in Lupiro village [3].

To conduct HLC, male volunteers sat with their legs exposed and caught mosquitoes that came to bite them with an aspirator [25]. Mosquitoes were caught for 45 min each hour, allowing 15 min break for rest. Catches were conducted between 19.00 and 07.00 hrs. The catches for each hourly interval were stored in separate collection cups.

The experimental details for the 1997 and 2004 surveys are described in Killeen *et al *[3]. Regarding the 2009 survey, each replicate collection of mosquitoes was made from an experimental unit that consisted of one experimental hut (simulating an average home) [26] and one outdoor sampling station situated 10 m from the hut. Each experimental hut contained two people and two untreated bed nets. Each experimental unit was separated by a distance of ≥30 m on flat land cleared of tall grass and other vegetation. One catcher caught mosquitoes in the outdoor station and another catcher simultaneously caught mosquitoes inside the experimental hut, the other person inside the hut was asleep under the bed net. There were four experimental units in total and on each night collections were made from one. Each night, the collectors were systematically rotated through the four of the experimental units using a Latin square design, to minimize biases due to individual odour or geographic location. The survey was conducted for 20 consecutive nights in June 2009.

All anopheline mosquitoes were morphologically identified to sex and species or species complex then visually classified as being unfed, partially fed, fully fed or gravid [27,28]. Throughout the 2009 survey, sub-samples of up to nine individual mosquitoes were taken from each trap to determine sibling species identity within the *An. gambiae *s.l. complex using PCR [29]. Prior to molecular analysis, individual mosquitoes were stored at -20°C in micro-centrifuge tubes containing a small amount of silica drying agent separated from the mosquito by a thin layer of cotton. For statistical analysis, a longitudinal dataset of *An. gambiae *s.l. sibling species composition was constructed for Lupiro village using all published literature [19,26,30-34] as well as unpublished data held at the Ifakara Health Institute (IHI). The small numbers of *An. funestus *complex mosquitoes caught during the 2009 data collection were discarded; however, subsequent indoor catches in the same experimental huts obtained specimens which were used to determine the sibling species composition of this complex in Lupiro immediately after that period using PCR [35].

### Human behavioural surveys

The behaviour of the human population during night times was estimated from answers to questionnaires, collected from 398 households between 2002 and 2004 [3]. The household members were asked what time they usually went to bed and arose in the morning.

### Estimating proportion of human contact with malaria vector bites occurring indoors

The behavioural characteristics of the vector populations were compared using two entomological parameters: propensity to bite indoors (referred to as endophagy) and propensity to bite during the night when people usually sleep (referred to as nocturnality). Endophagy was calculated as the proportion of mosquitoes biting indoors as follows: I_18→06 hrs _/(I_18→06 hrs _+ O_18→06 hrs_); where I = the total number of mosquitoes caught indoors, O = the total number of mosquitoes caught outdoors and the subscripts represent the start time for each hour [36]. Nocturnality was calculated as the proportion of mosquitoes biting either indoors or outdoors during peak sleeping hours (hours starting 9 pm to 5 am) as follows: (I_21→05 hrs _+ O_21→05 hrs_)/(I_18→06 hrs _+ O_18→06 hrs_) [36]. Additionally, the proportion of human contact with mosquito bites occurring indoors (π_i_) was calculated by taking into consideration the movement pattern of people using two methods: (A) by weighting the mean indoor and outdoor biting rates throughout the night by the proportion of humans that are typically indoors or outdoors at each time period: *π_i _*= ∑[*I_t_S_t_*]/∑[*O_t_*(1-*S_t_*)] + *I_t_S_t_*; where S = the proportion of humans indoors [see reference 3 for more detail] and (B) by using a simple binomial formula which assumes all humans go indoors at exactly 2100 hrs and leave the house again at 0500 hrs: π_i _= I_21→05 hrs _/(I_21→05 hrs _+ O_18,19,20,06 hrs_) [36].

### Statistical analysis

Statistical changes in endophagy, nocturnal activity and the binomial estimate of human contact were compared over time using generalized linear mixed models (GLMM) with a binomial distribution, a categorical explanatory variable for study year and random factors for household nested within date. For each dependent factor, a binary dataset was constructed (using the formulas detailed above) to contain the number of mosquito bites (bites per person per night [b/p/n]) that occurred indoors and outdoors for each date × household combination. Longitudinal statistical changes in *An. gambiae *s.l. sibling species composition (binary dependent variable) were analysed using a generalized linear model (GLM) with a binomial distribution and explanatory factors for year and rainfall. Rainfall was incorporated to account for the possibility that climate change had altered long-term precipitation patterns; this could be an important confounder as *An. arabiensis *tends to be associated with relatively drier habitats [37]. The longitudinal rainfall data (2002-2009) was obtained from the nearby Kilombero Agricultural Training and Research Institute. All analyses were conducted using *R*, ver.2.9.1 [38].

### Ethics

Ethical approval for the study was obtained from the IHI Institutional Review Board (IHI/IRB/No. A50), the Medical Research Coordination Committee of the National Institute for Medical Research (NIMR/HQ/R.8a/Vol. IX/801) in Tanzania, and the Liverpool School of Tropical Medicine (09.60). Before the study commenced, written permission was obtained from each volunteer, who was informed, orally and via provision of a pamphlet about the potential risks and benefits of participating. After consenting, each volunteer was screened for malaria infection using microscopy and only malaria-free individuals were allowed to participate. All volunteers were administered daily prophylaxis (Malarone^®^, 250 mg atovaquone and 100 mg proguanil hydrochloride, GlaxoSmithKline) to prevent malaria infection during the course of the experiment. In addition, ready access to diagnosis and, if necessary, treatment (Coartem^®^, 80 mg artemether and 480 mg lumefantrine over three days, Novartis Pharmaceuticals) was provided throughout the study.

## Results

**Figure 2:**
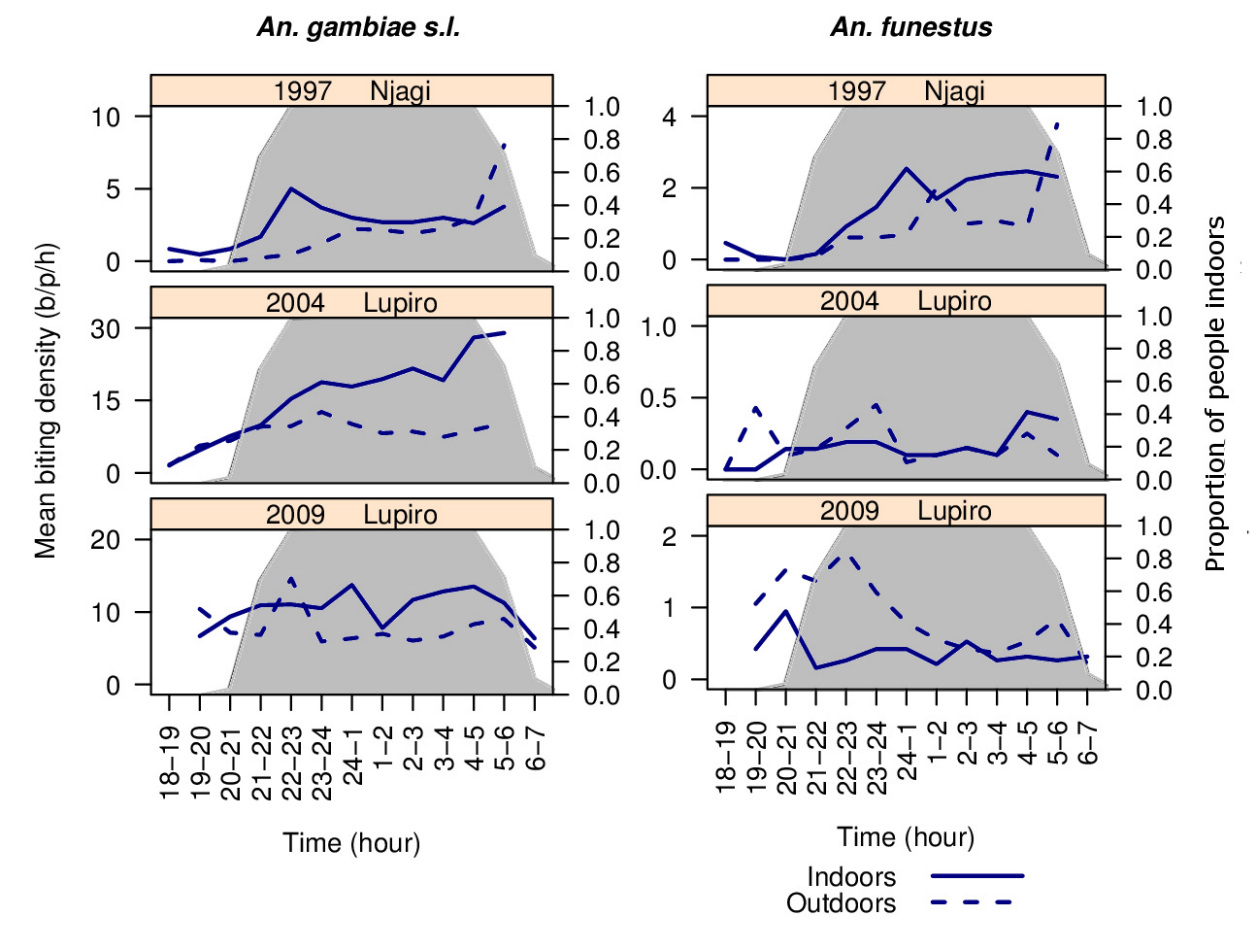
**The hourly indoor and outdoor biting profile of *Anopheles gambiae s.l*. (A - C) and *Anopheles funestus *(D - F) in the Kilombero Valley, Tanzania during 1997, 2004 and 2009**. The grey shading represents the proportion of the human population indoors. The value of 1 was added to each mean to avoid zero values for presentation using a log scale.

By 2009, after 47% use of long-lasting insecticide-treated nets, the biting profiles of *An. gambiae *s.l. and the *An. funestus *group had diverged from those observed 12 years earlier (Figure 2; Table 1). Regarding *An. gambiae *s.l. in 1997 this species exhibited a tendency to bite inside houses, late at night. By 2009, the proportion biting indoors (b/p/n) was not significantly different at 57.5% (Table 1). However, their nocturnal activity was significantly less and consistent activity was observed throughout the night (Figure 2; Table 1). Regarding *An. funestus*, in 1997 this species exhibited endophagic and nocturnal behaviour. By 2009, the tendency for *An. funestus *to bite indoors had significantly disappeared with less biting indoors than outdoors (Table 1). At this time, the nocturnality of *An. funestus *had also reduced (Table 1) and a peak in biting activity was recorded outdoors and early in the evening (Figure 2).

**Table 1:** The proportion (bites per person per night) of mosquitoes caught indoors and during sleeping hours during 1997, 2004 and 2009.

Year	**Proportion ± s.e**.	n/N^a, b^	Odds ratio [95% CI]	*p *value
***Endophagy*^a^**				
***Anopheles gambiae s.l***.				
1997^c^	0.585 ± 0.019	394/674	1.00	NA
2004	0.660 ± 0.006	3,916/5,931	1.113 [0.887 - 1.395]	0.354
2009	0.575 ± 0.008	2,390/4,160	0.970 [0.770 - 1.221]	0.796
Overall influence of Year		6,700/10,765	NA	0.248
***Anopheles funestus ***				
1997^c^	0.608 ± 0.025	217/357	1.00	NA
2004	0.463 ± 0.055	38/82	0.726 [0.461 - 1.142]	0.166
2009	0.298 ± 0.027	86/288	0.455 [0.323 - 0.641]	<0.0001
Overall influence of Year		341/727	NA	0.0001
***Nocturnality*^b^**				
***Anopheles gambiae s.l***.				
1997^c^	0.957 ± 0.008	645/674	1.00	NA
2004	0.902 ± 0.004	5,347/5,931	0.942 [0.840 - 1.056]	0.305
2009	0.794 ± 0.006	3,303/4,160	0.829 [0.738 - 0.933]	0.0018
Overall influence of Year		9,295/10,765	NA	<0.0001
***Anopheles funestus ***				
1997^c^	0.980 ± 0.007	350/357	1.00	NA
2004	0.829 ± 0.042	68/82	0.846 [0.594 - 1.208]	0.354
2009	0.704 ± 0.027	203/288	0.719 [0.570 - 0.907]	0.0054
Overall influence of Year		621/727	NA	0.0200

Proportions for each survey period are compared using GLMMs with a binomial distribution, a categorical explanatory variable for study year and a random factor for date.

^a ^Proportion of mosquitoes caught indoors calculated as: (I_18→06 hrs_)/(I_18→06 hrs _+ O_18→06 hrs_)

^b ^Proportion of mosquitoes caught during hours when most people are asleep calculated as: (I_21→05 hrs _+ O_21→05 hrs_)/(I_18→06 hrs _+ O_18→06 hrs_)

^c ^Formed the reference category for the GLMM

**Figure 3:**
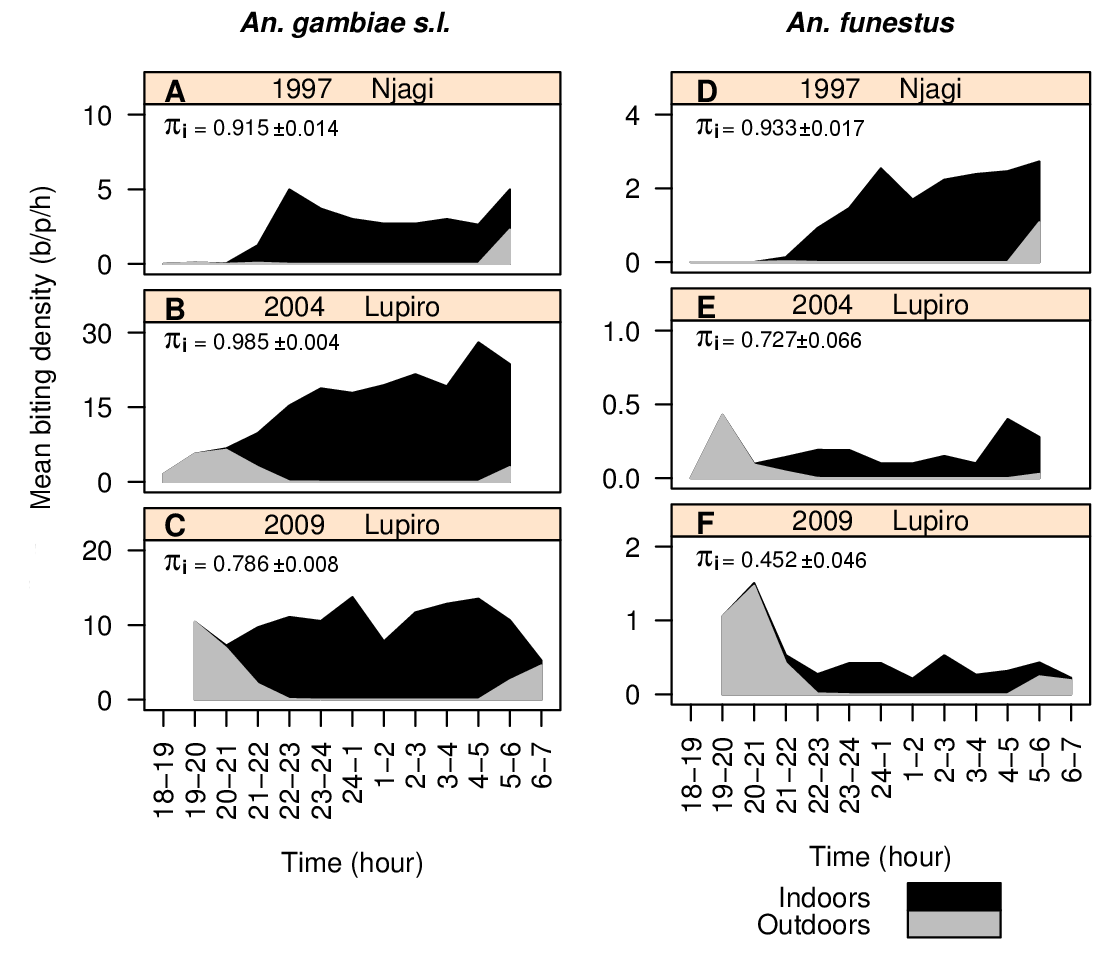
**The hourly indoor and outdoor profile of human contact with *Anopheles gambiae s.l*. (A - C) and *Anopheles funestus *(D - F) bites in the Kilombero Valley, Tanzania during 1997, 2004 and 2009**. This stacked line graph presents estimates of human indoor and outdoor contact rates, taking into consideration the movement pattern of people by weighting the mean indoor and outdoor biting rates throughout the night by the proportion of humans that are typically indoors or outdoors at each time period [3]. The value of 1 was added to each mean to avoid zero values for presentation using a log scale.

**Figure 4:**
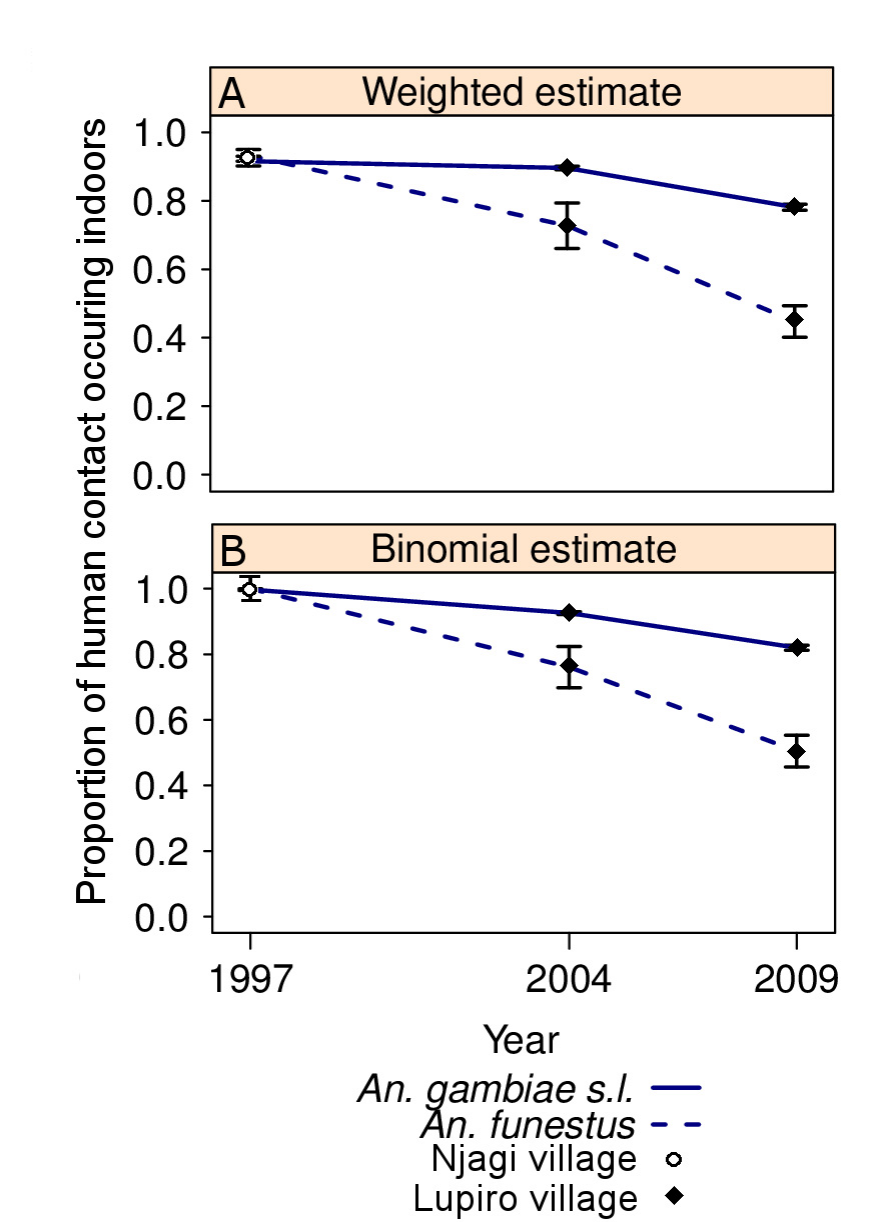
**Graphical comparison of the historical and recent estimates of the proportion of human contact with Anophelines occurring indoors (π_i_)**. The proportion of human contact with mosquito bites occurring indoor (π_i_) was calculated by taking into consideration the movement pattern of people using two methods: (A) by weighting the mean indoor and outdoor biting rates throughout the night by the proportion of humans that are typically indoors or outdoors at each time period and (B) using the formula: (I_21→05 hrs_)/(I_21→05 hrs _+ O_05→21 hrs_).

Most importantly, significant changes in the proportion of human contact with mosquito bites occurring indoors were recorded for both of these important vector taxa (Figure 3; Table 2). After adjusting for the typical movement of people, it was strikingly clear that after the introduction of ITNs, the proportion of human contact occurring indoors was reduced as contact occurring outdoors in the early evening proportionally increased (Figure 3). This change was evident in both taxa, but was more prominent for the populations of *An. funestus*. Regarding *An. gambiae *s.l. in 1997, the vast bulk of human contact to bites occurred when people were indoors (π_i _= 99.7% [se = 0.2]). After high coverage of untreated nets had been achieved by 2004, the proportion of indoor contact remained similarly high (π_i _= 92.6% [se = 0. 4]). After community-wide use of ITNs was achieved by 2009, the proportion of indoor contact (π_i_) with *An. gambiae *s.l. bites had dropped to 82.0% (se = 0.8) (Table 2, Figure 4). Regarding *An. funestus *in 1997, the vast bulk of human contact with bites also occurred with people were indoors (π_i _= 100% [se<0.1]). By 2004, the proportion of indoor contact (π_i_) had slightly, although not significantly, decreased to 76.1% (se = 6.3). By 2009, the proportion of indoor contact with *An. funestus *bites (π_i_) had dropped to only 50.5% (se = 4.8; Table 2 Figure 4). At this point in time, half of human contact with the *An. funestus *group was occurring outdoors, primarily before 9 pm (Figure 3). The estimates presented in Table 2 were calculated using the binary formula and are very similar to the more subtly calculated estimates (Figure 4) in which biting rates were weighted by the proportion of humans reporting to be indoors and outdoors at that time (Figures 3).

**Table 2:** The proportion of human contact with mosquito bites occurring indoors (π_i_) in the Kilombero Valley, Tanzania during 1997, 2004 and 2009.

Year	**Proportion ± s.e**.	n/N^a^	Odds ratio [95% CI]	*p *value
***Anopheles gambiae s.l***.				
1997^b^	0.997 ± 0.002	366/367	1.00	NA
2004	0.926 ± 0.004	3,622/3,912	0.928 [0.797 - 1.080]	0.337
2009	0.820 ± 0.008	1,964/2,395	0.822 [0.703 - 0.962]	0.0143
Overall influence of Year		5,952/6,674	NA	0.0019
***Anopheles funestus ***				
1997^b^	1.000 ± 0.000	210/210	1.00	NA
2004	0.761 ± 0.063	35/46	0.761 [0.471 - 1.229]	0.264
2009	0.505 ± 0.048	54/107	0.504 [0.345 - 0.737]	0.0004
Overall influence of Year		299/363	NA	0.0014

Proportions for each survey period are compared using GLMMs with a binomial distribution, a categorical explanatory variable for study year and a random factor for date.

^a ^Calculated as: (I_21→05 hrs_)/(I_21→05 hrs _+ O_18,19,20,06 hrs_)

^b ^Formed reference category for GLMM

**Figure 5:**
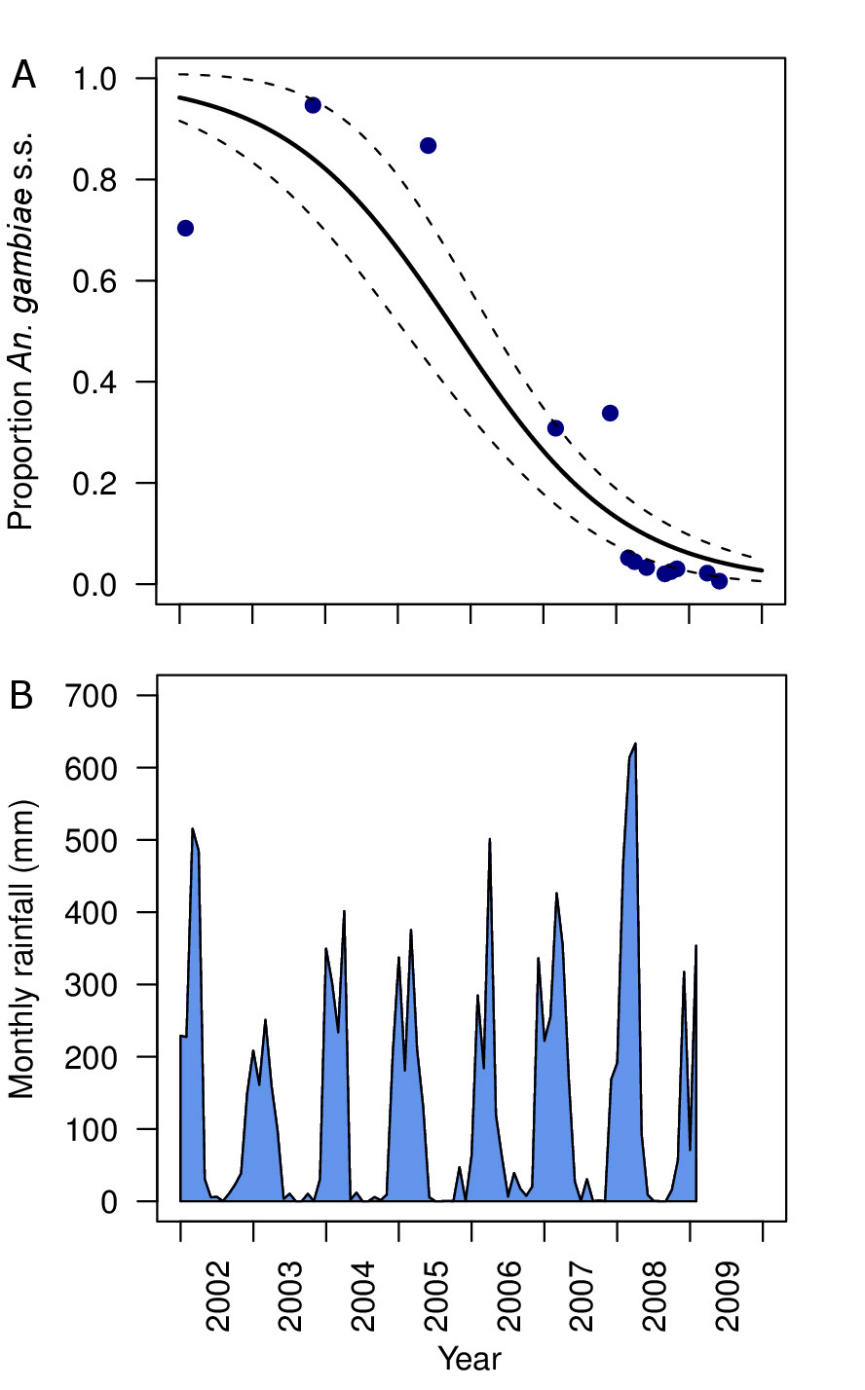
**The sibling species composition of the *Anopheles gambiae s.l*. complex (A) and monthly rainfall (B) between 2002 and 2009**. For (A), the plotted line represents the predicted fit (*β*) of a GLM with a binary distribution and a logistic link function (Solid line = fitted; Dashed lines = se). References: 2002: [19]; 2003: [30]; 2005: [31]; 2007: [26,32]; 2008: [33] and Moore et al. Unpublished data; 2009: [34] and current data.

*Anopheles gambiae *s.l. and the *An. funestus *group were by far the most important malaria vectors present during all survey years. Regarding *An. gambiae *s.l., the sibling species complex was predominated by *An. gambiae *s.s. in 1997 and 2004 [3]; however, by 2009 the sibling species composition had shifted to be predominated by *An. arabiensis *(99.5%; 849/854 of successful PCR amplifications). Only five individuals were positively identified as *An. gambiae *s.s. and these mosquitoes were all caught between 10 pm and 5 am. Although no longitudinal data were available for Njage village, a shift in *An. gambiae *s.l. sibling species composition has been observed in Lupiro (Figure 5). The longitudinal shift in sibling species composition towards *An. arabiensis *was statistically associated with year (β = -1.152, se = 0.038, p < 0.0001), but was not related to rainfall (climate) patters (β = -3.079 × 10^-4^, se = 2.044 × 10-4, p = 0.132). Unfortunately no historical information regarding the sibling species composition of the *An. funestus *group is available, but subsequent follow up surveys and successful PCR amplifications of 233 specimens in March 2010 confirmed that the vast majority (96.6%; n = 225) of these were *An. funestus *sensu stricto with the small remainder being *An. rivolurum *(3.4%; Okumu et al. Unpublished data).

## Discussion

These observations from a previously hyper-endemic setting demonstrate that community-wide ITN use can alter vector populations and reduce the epidemiological importance of indoor-biting mosquitoes. This is consistent with the knowledge that ITNs can reduce the mean density, survival, infectiousness and fitness of mosquito populations [6-9] as a direct function of the proportion of human contact to the vector occurring indoors [36,39]. This analysis was conducted retrospectively, based on the fortuitous availability of complementary data from unrelated studies over the years, rather than datasets collected and specifically tailored to examine trends in both the absolute intensity of transmission and the proportions of it which occurred indoors and outdoors. Unfortunately, no longitudinal, year-round, demographically and representatively sampled surveys of human biting mosquitoes in these two specific villages are available from which measures of absolute EIR could be estimated. However, it is likely most that the consistently high ITN use across the entire Kilombero Valley [19] had achieved similarly impressive reductions of EIR in Njage and Lupiro to those observed in the nearby villages of Idete and Namawala where detailed longitudinal entomological studies have been undertaken since 1990 [22]. Thus, the observations in the current study refer to one of the key mechanisms by which residual transmission could be maintained in communities using ITNs where the overall transmission intensity has been dramatically reduced. It is important to note that, the observed changes in feeding patterns are a consequence of killing vector mosquitoes, should not undermine confidence in ITN use, as they are an indicator of successful control. This is supported by recent theoretical models which demonstrate that an attenuated, but nevertheless valuable, amount of personal plus communal protection is provided by ITNs even when the proportion of contact that occurs indoors drops to 50%, as reported here for *An. funestus *[36]. Nonetheless, in such situations, any residual transmission will be predominantly maintained by a population of mosquitoes that, biting outdoors at dusk and dawn, may respond poorly to further measures targeted inside houses. The existence of shifting feeding patterns obviously hinders efforts to eliminate malaria with current proven methods, but also reflects encouraging success where the impact that can be obtained with ITNs or IRS is pushed to the limits of what is realistically achievable [39].

It was not possible to contrast these results with a comparison site without ITNs, and such a study would be ethically inappropriate in Tanzania or any other country where ITN access and use is rapidly increasing. Nevertheless, a very plausible case [40] is presented that correlates community-wide ITN use with significant changes in the biting profile of the principal malaria vectors. The human-biting behaviour of vectors in this part of Africa appears to independent of population density for these species [41]. This indicates that the observed variations in density, which are natural fluctuations due to seasonality or locality, would have had minimal bias on the observed shifts in behaviour. Although other environmental and anthropogenic factors may have influenced the mosquito biting behaviour, the differences recorded over time were significant enough that even mild confounding is unlikely to change the overall conclusions. In the current study, the longitudinal influence of rainfall on sibling species composition was quantified and it was observed that climate had not been significantly altered during the study period. Possible anthropogenic confounders include changes in land-use or human behaviour. With time, the population and geographic size of the villages did increase. However, the times at which the population entered and exited houses for sleep during the night remained similar.

Shifts in the sibling species composition are the most likely factor contributing to the observed changes in biting patterns for *An. gambiae *s.l. The use of ITNs in the study area [Figure 5, [22]] and in other contemporary settings [42] has resulted in a more dramatic drop in the density of highly anthropophagic and endophagic *An. gambiae *s.s. relative to the zoophagic and adaptable *An. arabiensis*. Regarding the *An. funestus *group, *An. funestus *s.s. was still the predominant species. However, historical reports from IRS campaigns suggest that for this species group, true species replacement [43] can occur with highly anthropophagic, endophagic *An. funestus *s.s. being replaced by *Anopheles parensis *or *Anopheles rivulorum*, which are far less potent vectors [28,44,45]. Contemporary examples of changing vector population composition resulting from widespread ITN use in rural Tanzania and Kenya [22,42], and similar historical observations associated with IRS [28,43-45], suggest that such shifts in vector composition may increasingly become the rule rather than the exception for African communities as coverage with one, or both, of these measures increases.

Regarding *An. funestus *s.s., density changes and/or behavioural avoidance could underlie the biting time shifts of this species. ITN use creates a stressful environment that has reduced the density of indoor biting mosquitoes, which in turn could lead to selection of resistant phenotypes [46]. Prolonged and wide-spread use of ITNs could, thereby, favour traits such as biting outdoors or early in the evening; these traits may be expressed by way of phenotypic plasticity or if these traits have a genetic basis, they may be selected to increase in frequency in a population [46]. The selection of behavioural traits is difficult to detect, but changes in mosquito biting behaviour have been shown to be immediately and directly induced by vector control tools, especially when excito-repellent insecticides are used [3].

As indoor interventions successfully eliminate the mosquitoes responsible for the majority of transmission, secondary sources of transmission-i.e. outdoor biting mosquitoes-will become culpable for a greater proportion of the declining overall rate of human infections than before. These outdoor-biting mosquitoes respond poorly to further insecticidal measures within houses. Additional vector control tools which target outdoor biting mosquitoes at the adult or immature stages are required to complement ITNs and IRS. This need for complementary tools and a reprioritisation of research funding is supported by recent reviews [47] and models [39] suggesting that intra-domicilary tools alone are insufficient to drive the parasite prevalence towards elimination in much of the malarious tropics, Africa in particular. Complementary tools, such as repellents [48,49], larval control [50,51] or zooprophylaxis [52], might be used to further suppress malaria transmission by providing personal-protection or reducing the survival, fitness and transmission potential of vector populations.

## Abbreviations

ITN: insecticide treated net; IRS: indoor residual spraying; HLC: human landing catch; GLMM: generalized linear mixed model; π_i_: proportion of human contact to mosquito bites occurring indoor; IHI: Ifakara Health Institute.

## Competing interests

The authors declare that they have no competing interests.

## Authors' contributions

Conceived and designed the experiments: GFK (2004 and 2009), CJD (1997). Performed the experiments: TLR, NJG. Analysed the data and wrote the manuscript: TLR, GFK. Reviewed the manuscript: NJG, CJD, SPK. All authors have read and approved the final manuscript.
